# Temporomandibular joint ankylosis

**Published:** 2009

**Authors:** Mukund Jagannathan

**Affiliations:** Department of Plastic, Reconstructive and Aesthetic Surgery, LTMG Hospital, Sion, Mumbai, India

Temporomandibular joint (TMJ) ankylosis, is fairly commonly seen and its treatment objectives are fairly well standardised, viz:

- Release/ resection of the bony mass
- Release of all restrictive forces preventing the opening of the jaw
- Allow for normal or near normal movements of the TMJ
- Ensure maintenance of the gap created, both to prevent reankylosis and to maintain vertical height of the mandible
- Promote growth and correct subsequent occlusal and cosmetic deformities

The authors have correctly pointed out that gap arthroplasty is more or less outdated. There are very few studies showing favourable outcomes.[1] It runs a high risk of re-ankylosis, and does not achieve many of the aims of surgery. Interposition arthroplasty seems to be the mainstay of treatment. The nature of the interposition is what needs to be discussed. An ideal interposition would fulfil the following criteria:

- Autogenous tissue
- Available in the vicinity, i. e. same operative field
- Should not change in volume and characteristics over time
- Should promote growth
- Should recreate as close to a normal joint as possible.

As yet, there is no single modality which meets all these requirements.

The authors have used three types of interpositional material. What they have not mentioned is the basis for selection of each, in a given case. The roles of silastic interposition as well as temporalis fascia are similar, but the costochondral graft has a totally different purpose. These purposes are not interchangeable, and each has clear cut indications. Costochondral grafts are used in the growing mandible, as an adaptive growth source.

The etymology of flaps used needs to be standardised. The authors refer to temporal fascia and temporalis fascia in different places in the article. A more appropriate term would be deep temporal fascia, which is the deep fascia overlying the temporalis muscle. It is not clear if or how the fascia was maintained in the gap. There is no mention of any retaining sutures. The thickness of even folded fascia, is still less than the extent of bony resection.

The duration of ankyloses has not been mentioned by the authors. It is relevant, and plays a great role in the eventual successful rehabilitation of the patient.

Though the authors have obtained the most satisfactory results in terms of mouth opening and non-recurrence of the ankylosis, it would be unfair to conclude unequivocally that deep temporal fascia is the best interposition material. The variables are several, including extent of ankylosis, duration, mouth opening on table, compliance of the patient in the postoperative exercise regimen, etc. Again, a year's follow up may not take into account growth changes which are age related.

Interposition arthroplasty, over a long term follow up should maintain the gap consistently. This has to be studied by imaging including CT and MR, to determine the integrity of the joint space.

Unfortunately, most of the locally available tissues (muscle, temporoparietal, as well as deep temporal fasciae) even though vascularised, tend to atrophy over a period of time. In all operated cases of re-ankyloses, there is no evidence of the nature of the tissue used.

Cartilage disc interposition has been well studied in sheep models, and show consistent appearance of pseudo joint spaces above the cartilage.[2] The cartilage itself undergoes organisation into an organised structure. Even masseter muscle transposition has been studied in experimental animals, and the muscle histology has revealed fairly viable muscle, with partial fibrosis, which maintains a good interposition.[3] There have been no studies for histological or long term radiological integrity of a fascial flap. Vascularised cartilage, as used by this author (Mukund Jagannathan) and reported in a previous issue of this journal^4^, appears to fulfil several of the criteria. Long term follow up images will soon be in print.

To summarise, interposition arthroplasty is part of the surgical treatment of TMJ ankylosis. This can be performed with several tissues. It is difficult to conclude that one is clearly better than the other, since the indications for each would vary. Long term detailed evaluation is needed before any conclusions can be drawn.
